# G9a: An Emerging Epigenetic Target for Melanoma Therapy

**DOI:** 10.3390/epigenomes5040023

**Published:** 2021-10-12

**Authors:** Jessica L. Flesher, David E. Fisher

**Affiliations:** Cutaneous Biology Research Center, Department of Dermatology, Massachusetts General Hospital, Harvard Medical School, Charlestown, MA 02129, USA;

**Keywords:** G9a, epigenetic inhibitors, melanoma, EZH2

## Abstract

Epigenetic regulation is a crucial component of DNA maintenance and cellular identity. As our understanding of the vast array of proteins that contribute to chromatin accessibility has advanced, the role of epigenetic remodelers in disease has become more apparent. G9a is a histone methyltransferase that contributes to immune cell differentiation and function, neuronal development, and has been implicated in diseases, including cancer. In melanoma, recurrent mutations and amplifications of G9a have led to its identification as a therapeutic target. The pathways that are regulated by G9a provide an insight into relevant biomarkers for patient stratification. Future work is aided by the breadth of literature on G9a function during normal differentiation and development, along with similarities to EZH2, another histone methyltransferase that forms a synthetic lethal relationship with members of the SWI/SNF complex in certain cancers. Here, we review the literature on G9a, its role in melanoma, and lessons from EZH2 inhibitor studies.

## 1. Epigenetic Regulation by Histone Methyltransferases

While DNA encodes all the necessary genes, the reversible modifications to DNA and histone complexes allows for one individual to undergo development from a zygote to a complex multi-cellular organism. Open chromatin that is permissive for transcription is often marked with nearby histone acetylation. Depending on the context, histone methylation can provide active marks, such as histone 3 lysine 4 (H3K4), but are more often associated with repression through H3K9 and H3K27 methylation found near compacted chromatin that is inaccessible for transcription [1]. Additional modifications to histones include phosphorylation, ubiquitination, and sumoylation, which alter transcription and repair, while DNA can be modified through methylation to alter transcription [2]. Together, histone modifications and DNA methylation tightly regulate chromatin accessibility within cells.

**Figure 1.**
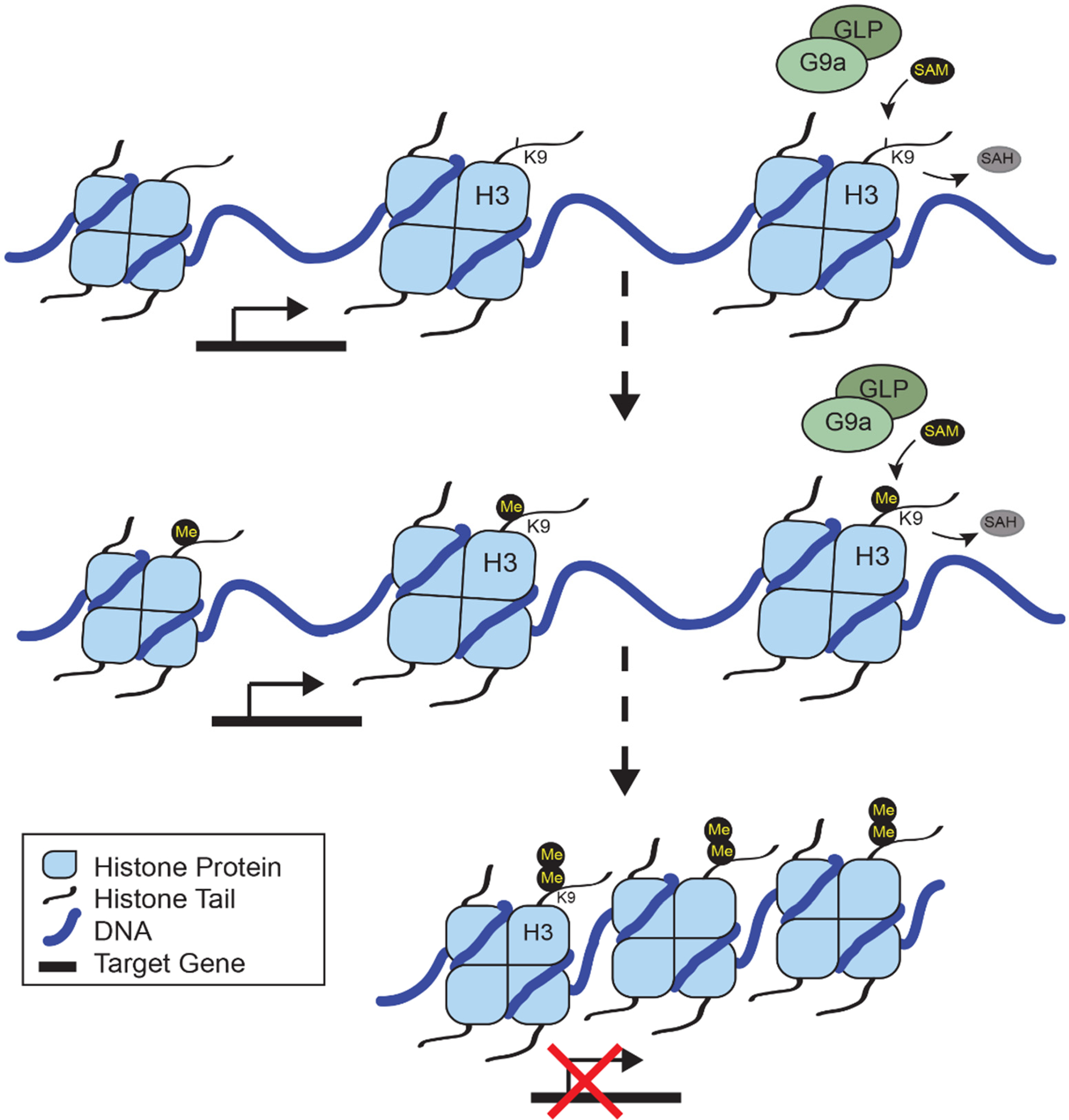
G9a-dependent methylation of H3K9 leads to transcriptional repression. G9a forms a dimer with GLP which recognizes histone 3 lysine 9 (H3K9) for methylation. Using SAM (s-adenosyl-methionine) as the methyl donor, G9a transfers a methyl group (Me) to generate H3K9 monomethylation (H3K9me), releasing SAH (s-adenosyl homocysteine). G9a/GLP dimers further methylate H3K9me to generate dimethylation (H3K9me2). At sites where H3K9me2 is present, chromatin is compacted leading to transcriptional repression of target genes.

Histone methyltransferases are members of a large family of proteins that require the methyl group donor s-adenosyl-methionine (SAM) to methylate specific residues on the histone tail, or add to previously methylated residues to form di- and trimethylation. Histone 3 lysine 9 (H3K9) is initially methylated by G9a (*EHMT2* gene) and GLP (*EHMT1* gene) dimers to form activating H3K9 monomethylation, and it is further methylated by G9a to form repressive H3K9 dimethylation (H3K9me2) [3], as illustrated in Figure 1. Both G9a and GLP contain ankyrin repeats to recognize these methylation residues, and an SET domain that is required for catalytic activity that utilizes SAM. The G9a/GLP complex is required for methyltransferase activity, since a loss of either G9a or GLP can significantly reduce H3K9me2 in the brain [4]. While there is some evidence that G9a may facilitate the trimethylation of H3K9, the histone methyltransferase SETDB1 is critical for maintaining the DNA methylation of retrotransposons through H3K9me3 [5], while SUV39H1 and SUV39H2 deposit the trimethylation of H3K9 at constitutive heterochromatins [6]. This means that G9a directly contributes to repressive dimethylation and subsequent trimethylation at H3K9 throughout the genome.

Though histone methyltransferases are thought to function specifically in histone regulation, G9a can also methylate proteins with histone methylation site-like residues. G9a itself has an automethylation site near the N-terminus that increases functional activity and interaction with HP1, a protein enriched in euchromatin [7]. G9a can methylate additional non-histone proteins in complex with GLP, or alone, modulating proteins including WIZ, a binding partner of G9a; HIF-1alpha, blocking the transcription activity of hypoxia target genes; p53, a key tumor suppressor; and HDAC1 and DMNT1, a histone deacetylase and DNA methyltransferase, respectively [8–10]. The number of G9a targets and its repressive role in gene expression both directly and indirectly showcase the impact that epigenetic regulators can have on a cell.

## 2. The Role of G9a in Development and Differentiation

During development and differentiation, cells actively open and close chromatin to turn off genes that were required for earlier states and turn on gene programs that facilitate proper differentiation. H3K9me2 is required during early embryonic development, since the constitutive loss of *Ehmt2* causes embryonic lethality at 11.5 days and loss of the maternal copy alters DNA methylation during zygote development [11]. G9a also contributes to silencing *Oct3*/*4* and *Nanog* during early embryonic development [9]. In embryonic stem cells, G9a has been implicated in the regulation of large organized chromatin K9 modifications [12], but additional analyses are necessary to validate the findings, as described by Filion and Steensel [13]. Altogether, G9a is required during development and represses key genes to allow for the differentiation of cell types.

G9a is also an important regulator of immune cell-type specific regulation. G9a and other methyltransferases regulate T cell fate decisions, as well as other lymphoid cells [14,15]. The transition from T-helper cells into functional CD8+ T cells relies on G9a to maintain the initial repression of *CD4* by RUNX3, so only the long-term maintenance of chromatin marks is effected by G9a loss [16]. The transcription factor BLIMP-1 also recruits G9a to promote CD8+ memory T cell terminal differentiation, by repressing *Il2ra* and *Cd27* to stimulate an anti-viral response [17]. In B cells, G9a loss does not inhibit differentiation, but may affect the stability of mature B cells [18]. Additional evidence identified MHC class I proteins as targets for G9a repression [19]. Proteins that interact with G9a, including JARID2, also contribute to normal differentiation of additional immune cells, such as invariant natural killer T cells (iNKT) that are derived from CD4/CD8 double-positive T cells [20]. As an important regulator of immune differentiation, G9a contributes to both normal differentiation and the response to disease.

## 3. The Oncogenic Roles of G9a in Melanoma

Recent publications have identified G9a as a functional oncogene in melanoma, through recurrent activating mutations in the SET domain or through amplification of the genomic locus [21]. Some key pathways in melanoma are perturbed by G9a amplification, as summarized in Table 1. Wnt ligand signaling leads to a cellular cascade that prevents beta-catenin destruction allowing nuclear translocation in order to bind and promote transcription factors, such as LEF1. In melanocytes, the melanoma cell of origin, this process contributes to pigmentation, since several genes that are required for melanin synthesis are bound and transcriptionally activated via LEF1 [22]. Studies on Wnt signaling in melanoma have found that the stabilization of beta-catenin promotes tumor development and inhibits the response to immunotherapy in *Braf*^*V600E*^ mice with a loss of *Pten* [23,24]. Using the same melanoma model, the knockout of beta-catenin, which blocks Wnt signaling, inhibits tumor formation [23]. Wnt signaling can also be blocked through repressors, including DKK1, triggering beta-catenin degradation by the deconstruction complex, leading to the repression of Wnt target genes. *DKK1* was found to be repressed by G9a overexpression in immortalized primary melanocytes, as well as upregulated when G9a was knocked down in melanoma cells [21]. While human melanoma in the TCGA shows a reverse relationship between G9a expression levels and immune signature genes, mouse studies in melanoma found that G9a inhibition, in combination with anti-PD-1 or anti-CTLA-4, increased survival compared to immunotherapy alone [21]. In neuroendocrine tumors, a loss of G9a or inhibition of H3K9me2 leads to the upregulation of *DKK1*/*3* [25]. Similarly, in colorectal cancer, G9a and H3K9me2 marks are enriched at multiple known negative regulators of Wnt signaling, using patient-derived organoids [26]. Together, these studies highlight a possible mechanism through which G9a inhibition could modulate Wnt signaling, to both directly repress melanoma cell oncogenic behavior and increase responses to immunotherapy.

Another pathway that is altered by G9a inhibition and knockdown is Notch signaling [27]. Notch signaling relies on ligand binding to the notch receptor, leading to intracellular cleavage of the NICD domain that functions as a transcription factor for target genes. In melanoma, elevated Notch signaling has been linked to antitumor immunity [28], resistance to MEK inhibitors [29], and repression of MITF, leading to an invasive phenotype [30]. Notch signaling can also impact G9a, since an NICD-induced model of cholangiocarcinoma, a cancer arising in the bile duct, led to an increase in G9a levels, and subsequent tumors were sensitive to G9a inhibition [31]. Blocking Notch signaling within tumor cells may promote a less differentiated, treatment-resistant tumor; however, the inhibition of Notch signaling may not be entirely straight forward. Some evidence suggests that in melanoma-associated fibroblasts, Notch signaling decreases cell viability, creating a less favorable tumor microenvironment for the melanoma [32], and the loss of Notch signaling in fibroblasts can promote melanoma-initiating cells [33]. As with any targeting strategy, the possible benefits of G9a inhibition on tumor cells and the immune response will have to be weighed against the negative impacts on cancer-associated fibroblasts or other tumor microenvironment factors.

G9a has also been linked to tumor growth and metastasis. In vitro studies using serial detachment and re-adhesion of Melan-A cells also found that H3K9me2 increased between non-metastatic mesenchymal-like melanoma cells, compared to the metastatic cells [34]. The detachment and re-adhesion of cells on a plate is a highly artificial model of epithelial-to-mesenchymal transition, but additional evidence for a role of G9a in melanoma metastasis can be found in other tumor types. In glioblastoma, G9a, along with other methyltransferases, can be sequestered by the lncRNA HOTAIRM1, which allows for the upregulation of *HOXA1* to promote tumor growth [36]. The HOX genes are known to play a role in melanoma, with *HOXA1* upregulation being indicative of highly metastatic tumors [35]. Additional evidence in breast cancer has shown that, during hypoxia, G9a represses *CDH10*, a cell adhesion molecule, increasing cellular motility [37]. Since G9a can have activities associated with either promoting or suppressing metastatic behaviors, additional work is needed to assess whether G9a truly promotes metastasis through methylation, or if it acts as a repressor to HOX genes in melanoma, and whether these distinct activities may be dependent upon the context of other genomic aberrations within tumor cells, or perhaps within the microenvironment of the tumor.

Autophagy is another pathway that is perturbed by the modulation of G9a. In glioblastoma, G9a deficiency triggers autophagy and blocks proliferation through methylation of the *c-MYC* and *MAP1LC3B1* promoters [39,40]. Similar findings have been found in cervical and pancreatic cell lines, where G9a is ejected from promoters of autophagy-related genes, including *MAP1LC3B*, under starvation conditions [41]. Additionally, G9a also blocks autophagy through H3K9me of the *mTOR* promoter in gastric cancer [42]. Though there is some evidence that G9a inhibition leads to the activation of autophagy through *MAP1LC3B* in melanoma [38], a more thorough assessment needs to be considered. Not only can autophagy be alternatively tumor suppressive or oncogenic, but additional parameters can conflate the role of G9a. Careful consideration must be given to factors including the concentration range of G9a inhibitors for optimal dosing and the timing required to induce long-term epigenetic alterations in chromatin accessibility, since short-term effects may not persist, as observed with the G9a lost in immune cells. It is also highly likely that the alternative consequences of autophagy modulation may be dependent upon the genomic context (including G9a status, as well as multiple others that remain to be identified).

As our understanding of G9a in melanoma continues to develop, it is apparent that the removal of repressive H3K9me2 can have wide-ranging effects on multiple signaling pathways that can alter both tumors and the cells of the microenvironment. Within the field of melanoma epigenetics, there is much room for further study, to dissect additional pathways that can be leveraged to identify patients that may benefit from G9a inhibition. Additionally, understanding the full impact of G9a inhibition in combination with current melanoma therapies will be important moving forward.

## 4. Targeting Histone Methyltransferases

Another point of reference, to aid in our understanding of the role of G9a in melanoma, is the array of literature on EZH2, which is the target of the first FDA approved histone methyltransferase inhibitor, tazemetostat, for the treatment of epithelioid sarcoma [43]. In the early 2000s, EZH2, which methylates H3K27, was shown to be upregulated in breast cancer, prostate cancer, and lymphoma [44–46]. In these studies, the increased EZH2 expression was higher in proliferating cells, associated with poor outcomes for patients, or higher in metastatic samples compared to localized tumors. As the field advanced, a synthetic lethal relationship was described with mutant ARID1A, a member of the SWI/SNF chromatin remodeling complex, in ovarian cancer, where EZH2 silences autophagy in the absence of functional ARID1A [47]. EZH2 inhibition may also be a feasible treatment in gastric cancer, where ARID1A mutant tumors often have high levels of EZH2 [48]. As a paralog of ARID1A, ARID1B inhibition in medulloblastoma can also lead to increases in H3K27 methylation [49]. ARID1B is also a dependency in ARID1A mutant ovarian tumors, where a loss of both mutually exclusive subunits decreases the overall stability of the SWI/SNF complex [50]; together, this suggests that both paralogs can form a synthetic lethal relationship with EZH2 inhibition. However, this relationship is not found in urothelial bladder cancer, where ARID1A regulates cell cycle regression rather than the repression of EZH2 [51], indicating that tumor types and subtypes may need to be assessed individually. Understanding the role of EZH2 is ongoing, but EZH2 as a cancer therapy is well under way, with additional clinical trials testing tazemetostat and other EZH2 inhibitors for additional sarcoma subtypes (NCT02601950, NCT02601937), lymphoid neoplasms (NCT03603951, NCT04407741), and in combination with BRAF and MEK inhibition in melanoma (NCT04557956). It will be of interest to determine whether any clinical efficacy that is observed shows a correlation with the status of SWI/SNF subunit mutations. Together, these studies and the ongoing clinical trials highlight the need for in-depth analysis of the mechanism through which G9a promotes melanoma growth, and whether additional epigenetic regulators contribute to this process.

G9a inhibitors are still in the early stages of development. Currently, three compounds have been widely utilized as G9a inhibitors, with both BIX01294 and UNC0638 used in vitro, and UNC0642 tested in preclinical models in vivo, due to its more favorable pharmacokinetics. Additionally, G9a targeted therapies may help a wider range of melanomas, since the amplification of G9a occurs in the context of both BRAF- and NRAS-activating mutations, which are the two most common oncogenic drivers of melanoma [21]. Multiple studies have also indicated that G9a inhibition combination treatment might boost the response of melanoma to immune checkpoint inhibition in mouse models [21,38]. While these responses could be indicative of tumor-specific inhibition, there is still more work to be conducted to characterize if G9a inhibition is synergistic to immune checkpoint therapies.

## 5. Conclusions

The many roles of G9a, from the repression of stem cell factors in early development and CD4 during CD8+ T cell differentiation to inhibiting the DKK1 repression of Wnt signal in melanoma, highlight the broad range of functions that are controlled by the epigenetic regulation of histone methylation. As an emerging potential druggable target, G9a has been implicated in direct epigenetic effects in melanoma, to alter both Wnt and Notch signaling, as well as contributing to pathways that promote metastasis. Though the full effects of G9a are still under investigation, there are abundant resources to direct future research on G9a interactions with other epigenetic regulators, through inhibition studies, RNA interference, CRISPR knockouts, and mouse models, while taking into account the broader impact of G9a inhibition on tumor-associated fibroblasts and the immune components of the microenvironment. The broad, genome-wide effects of epigenetic factors, such as G9a, highlight the challenges in seeking anti-tumor efficacy without excessive toxicity. Yet, the presence of tumors containing the “smoking gun” of G9a mutations or amplifications may increase the therapeutic index (efficacy vs. toxicity) and further support the opportunity to therapeutically antagonize this enzyme. As an emerging target for melanoma therapy, G9a is another example of the importance of epigenetic regulation in our understanding of tumor biology.

## Figures and Tables

**Table 1.** Signaling pathways and cellular mechanisms linked to G9a in melanoma.

Signaling Pathway/Cellular Mechanism	Key Findings	Tumor Type	Reference
Wnt Signaling	The Wnt repressor, DKK1, is targeted by G9a repression	Melanoma	[21]
	Wnt signaling promotes melanoma development and inhibits immunotherapy response	Melanoma	[23,24]
	G9a loss or inhibition of H3K9me2 causes upregulation of DKK1	Neuroendocrine	[25]
	G9a is enriched at genes that negatively regulate Wnt signaling in patient-derived organoids	Colorectal Cancer	[26]
Notch Signaling	Overexpression of G9a promotes upregulation of Notch1 signaling pathway	Melanoma	[27]
	Elevated Notch signaling associated with treatment resistance and more invasive phenotypes	Melanoma	[28–30]
	Notch signaling in fibroblasts creates a less favorable microenvironment	Melanoma	[32,33]
	G9a levels increase in NICD-induced model of bile duct cancer	Cholangiocarcinoma	[31]
Metastasis	H3K9me2 is increased between non-metastatic mesenchymal-like to metastatic melanoma cells	Melanoma	[34]
	HOX genes are upregulated in metastatic melanoma	Melanoma	[35]
	HOXA1 is a repressed target of G9a	Glioblastoma	[36]
	G9a represses CDH10 increasing cellular motility during hypoxia	Breast Cancer	[37]
Autophagy	G9a inhibition activates autophagy	Melanoma	[38]
	G9a represses MAP1LC3B and blocks autophagy	Glioblastoma	[39,40]
	G9a vacates autophagy related gene promoters under starvation in vitro	Cervical and Pancreatic Cancer	[41]
	G9a blocks autophagy through activation of mTOR	Gastric Cancer	[42]
